# Determination of Relative Weightings for Sacroiliac Joint Pathologies in the OMERACT Juvenile Arthritis Magnetic Resonance Imaging Sacroiliac Joint Score

**DOI:** 10.3390/jcm12072729

**Published:** 2023-04-06

**Authors:** Tarimobo M. Otobo, Mirkamal Tolend, Arthur B. Meyers, Iwona Sudol-Szopinska, Sayali Joshi, Jennifer Stimec, Nele Herregods, Jacob L. Jaremko, Shirley M. L. Tse, Nigil Haroon, Rahim Moineddin, Nikolay Tzaribachev, Simone Appenzeller, Michal Znajdek, Manuela Perez, Aloysius E. Ligha, Lennart Jans, Emilio J. Inarejos Clemente, Pamela Weiss, Olympia Papakonstantinou, Eva Kirkhus, Marion A. J. van Rossum, Dax G. Rumsey, John Carrino, Jonathan D. Akikusa, Philip G. Conaghan, Andrea S. Doria

**Affiliations:** 1 Department of Diagnostic Radiology, Hospital for SickKids, Toronto, ON M5G 0A4, Canada; 2 Department of Radiology and Medical Imaging, Cincinnati Children’s Hospital Medical Center, Cincinnati, OH 45229, USA; 3 Department of Radiology, National Institute of Geriatrics, Rheumatology and Rehabilitation, 02-637 Warsaw, Poland; 4 Division of Pediatric of Radiology, Universitair Ziekenhuis Ghent, 9000 Ghent, Belgium; 5 Department of Radiology, University of Alberta, Edmonton, AB T6G 2R3, Canada; 6 Division of Rheumatology, SickKids, Toronto, ON M5G 1X8, Canada; 7 Department of Rheumatology, Toronto Western Hospital, Toronto, ON M5T 2S8, Canada; 8 Department of Family Medicine, University of Toronto, Toronto, ON M5G 1V7, Canada; 9 Department of Pediatric Rheumatology, Medical Center Bad Bramstedt, 24576 Bramstedt, Germany; 10 Department of Rheumatology, Universidade Estadual de Campinas, São Paulo 13083-970, Brazil; 11 Department of Diagnostic Radiology, University of Toronto, Toronto, ON M5G 1V7, Canada; 12 Department of Anatomy, Niger Delta University, Amassoma 560103, Nigeria; 13 Department of Radiology, Hospital Sant Joan de Deu, 08950 Barcelona, Spain; 14 Department of Rheumatology, Children Hospital of Philadelphia, Philadelphia, PA 19104, USA; 15 Department of Radiology, National and Kapodistrian University of Athens, 157-72 Athens, Greece; 16 Department of Radiology, Oslo University Hospital, 0372 Oslo, Norway; 17 Amsterdam Rheumatology and Immunology Center, Read and Emma Children’s Hospital, Amsterdam University Medical Center, 1105 Amsterdam, The Netherlands; 18 Division of Rheumatology, Department of Pediatrics, University of Alberta, Edmonton, AB T6G 1C9, Canada; 19 Department of Radiology and Imaging, Hospital for Special Surgery, New York, NY 10021, USA; 20 Department of General Medicine, Rheumatology Service, Royal Children’s Hospital Melbourne, Melbourne, VIC 3052, Australia; 21 Department of Rheumatology, University of Leeds, Leeds LS7 4SA, UK

**Keywords:** OMERACT, JAMRIS-SIJ, juvenile idiopathic arthritis, MRI, outcome measure, face validity, 1000Minds, conjoint analysis

## Abstract

This study aims to determine the relative weights (point value) of items of the juvenile idiopathic arthritis magnetic resonance imaging-sacroiliac joint scoring system (JAMRIS-SIJ). An adaptive multicriteria decision analysis was performed using the 1000Minds web application to determine the relative weights of the items in the JAMRIS-SIJ inflammation and damage domains. Experts in imaging and rheumatology independently completed a conjoint analysis survey (CAS) to determine the point value of the measurement items of the JAMRIS-SIJ. Each CAS survey question asked the expert to compare two hypothetical patient profiles, which were otherwise similar but different at two items at a time, and to select which item showed a more severe stage of inflammation or osteochondral damage. In addition, experts ranked 14 JAMRIS-SIJ grade only or image + grade patient vignettes while blinded to the CAS-derived weights. The validity of the weighted JAMRIS-SIJ was tested by comparing the expert CAS-weighted score and the image + grade ranking method. Seventeen experts completed the CAS (11 radiologists and 6 rheumatologists). Considering the point value for inflammation domain items, osteitis (24.7%) and bone marrow edema (24.3%) had higher group-averaged percentage weights compared to inflammation in erosion cavity (16.9%), joint space enhancement (13.1%), joint space fluid (9.1%), capsulitis (7.3%), and enthesitis (4.6%). Similarly, concerning the damage domain, ankylosis (41.3%) and erosion (25.1%) showed higher group-averaged weights compared to backfill (13.9%), sclerosis (10.7%), and fat metaplasia lesion (9.1%). The Spearman correlation coefficients of the CAS-weighted vignette order and unweighted JAMRIS-SIJ grade only order vignettes for all experts were 0.79 for inflammation and 0.80 for damage. The correlations of image vignettes among imaging experts to CAS were 0.75 for inflammation and 0.90 for damage. The multicriteria decision analysis identified differences in relative weights among the JAMRIS-SIJ measurement items. The determination of the relative weights provided expert-driven score scaling and face validity for the JAMRIS-SIJ, enabling the future evaluation of its longitudinal construct validity.

## 1. Introduction

Juvenile idiopathic arthritis (JIA) is a chronic arthritis of unknown etiology occurring before the age of 16 years. Sacroiliac joint (SIJ) pathology can most commonly be seen in the JIA subtype known as enthesitis-related arthritis (ERA). This can cause lower back, buttock pain, and stiffness in affected individuals [1]. JIA can significantly affect the psychosocial development and wellbeing of children with a substantial financial burden to health systems [2,3]. Commonly used assessment methods for JIA disease activity, such as clinical examination, patient-reported outcomes (PROs), and serological biomarkers, have variable reliability and validity [4]. Moreover, the anatomical location of the SIJ poses significant limitations for accurate clinical examination [5]. Early detection of SIJ inflammation in JIA is essential for therapeutic intervention to prevent disease progression and irreversible joint damage [6]. Radiography has been used for SIJ imaging in JIA, but it is not sensitive in detecting early inflammatory joint lesions [7,8]. Magnetic resonance imaging (MRI) is capable of detecting early SIJ pathologies, which is helpful for disease monitoring and treatment decision making in JIA [6,7]. However, there is a need for the standardization of SIJ MRI interpretation, and this need underpins the iterative development of the outcome measure in rheumatology (OMERACT) juvenile idiopathic arthritis MRI SIJ scoring system (JAMRIS-SIJ) as a standardized objective outcome measurement tool for the assessment of JIA treatment effectiveness in clinical trials [9].

The JAMRIS-SIJ is a multi-component outcome tool that semi-quantitatively measures inflammation and damage in the SIJ [9]. The component items (SIJ MRI pathologies) are distinct and have relative importance in measuring SIJ inflammation and damage. Each item contributes to part of the measurement construct, and in conjunction, the items form the entire construct. This conceptual framework between the measurement items and construct is a formative model [10]. Although the individual item scores can be meaningful when reported separately, it is also desirable to be able to aggregate the items in a domain to form a single composite score. This requires estimating the point value of the respective components for a formative model. Determining the relative weightings of the measurement components will provide a standardized and objective approach in deriving a composite domain score. In the absence of a feasible external criterion for SIJ inflammation and damage, multicriteria decision analysis (MCDA) (conjoint analysis) was utilized to elicit expert judgment to determine the relative weights of the measurement items [11]. A conjoint analysis survey elicited expert preferences for the relative importance of measurement items to define the relative weightings of the JAMRIS-SIJ [12,13].

This study aims to determine the relative weightings of the JAMRIS-SIJ measurement components as part of the face validity assessment of the OMERACT JAMRIS-SIJ.

## 2. Materials and Methods

We performed a partial profile conjoint analysis using a decision-making software application called 1000Minds to objectively elicit the opinion of imaging and rheumatology experts to determine the relative weights of the JAMRIS-SIJ. Afterwards, experts performed an independent ordinal ranking of 14 cases of JAMRIS-SIJ grade-only and image + grade-based vignettes, to test the face and convergent validity of the conjoint analysis derived-weighted JAMRIS-SIJ.

### 2.1. Conjoint Analysis Survey

**Figure A1:**
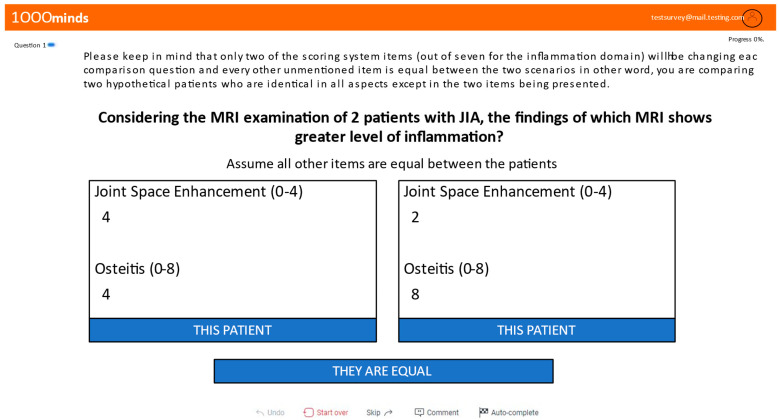
JAMRIS-SIJ inflammation domain item comparison. An example of an item comparison question presented by the 1000Minds software to the rating experts to elicit their preferences of the relative importance weights for the JAMRIS-SIJ inflammation domain items. The expert was asked to compare two hypothetical patients assuming all other features were equal: with a score of 4 each for joint space enhancement (JSE) and osteitis and patient; with a score of 2 for JSE and 8 for osteitis. The expert had to choose which patient showed greater inflammation or rate them as equal. The questions varied adaptively and were presented again until all necessary trade-off questions were completed by the expert to determine the relative weights for each domain.

**Figure A2:**
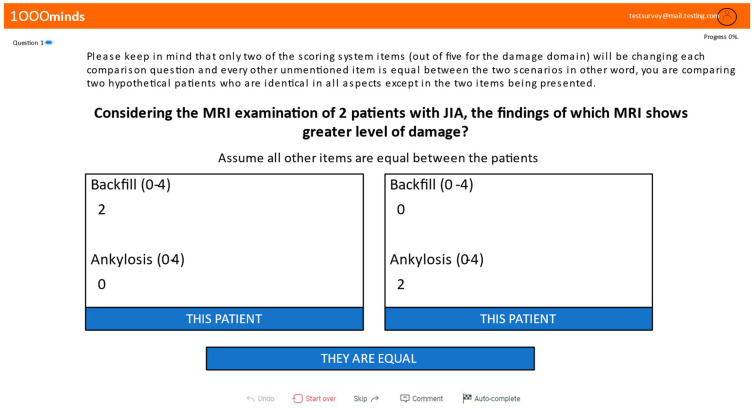
JAMRIS-SIJ damage domain item comparison. An example of an item comparison question presented by the 1000Minds software to the rating experts to elicit their preferences on the relative importance weights for the JAMRIS-SIJ damage domain items. The expert was asked to compare two hypothetical situations in patients with juvenile idiopathic arthritis assuming all other features were equal: with a score of 2 for backfill and 0 ankylosis and patient; with a score of 2 for ankylosis and 0 for osteitis. The expert had to choose which patient showed greater damage or to rate them as presenting with equal damage. The questions varied adaptively and were presented again until all necessary trade-off questions were completed by the expert to determine the relative weights for each domain.

The conjoint analysis survey (CAS) allowed seventeen experts comprising eleven radiologists (65%) and six rheumatologists (35%) to provide their preferences anonymously for each measurement item in the JAMRIS-SIJ to measure SIJ inflammation and damage in JIA. Over 80% of experts who completed the CAS had between 11–30 years of experience in imaging and rheumatology practice (Table S1). Experts were prompted to compare the measurement items (Figure A1 and Figure A2) conjointly, making trade-offs among the items according to their opinion of item importance using a CAS web application called 1000Minds [13].

The 1000Minds software utilized the ‘potentially all pairwise ranking of possible alternative’ (PAPRIKA) method to compute the relative weightings. In this method, the expert was required to pairwise rank potentially all pairs of possible alternatives of the JAMRIS-SIJ measurement items for each patient scenario. In each patient comparison scenario, a pair of hypothetical patients is presented to the expert, with each patient characterized by differing grades in two of the inflammation (Figure A1) or damage (Figure A2) domains of the JAMRIS-SIJ items. This hypothetical comparison scenario assumes all other JAMRIS-SIJ measurement items are equal for both patients. The pair of JAMRIS-SIJ measurement items presented to the expert for comparison (undominated pairs) constitute a partial profile of the JAMRIS-SIJ, as it is a partial set of the eight or five items of the two domains.

To complete the pairwise partial-profile comparison, the experts were instructed to choose the patient scenario which was greater in the level of inflammation or damage in the SIJ or rate them as equal. These comparison questions continue until the ranking of all possible alternative item combinations are determined adaptively based on the responses from the expert. The number of explicitly compared undominated pairs is reduced by the PAPRIKA method, which identifies and eliminates all pairs implicitly ranked as corollaries of the explicitly ranked pairs, using the transitivity property of additive multicriteria decision analysis [13].

To ensure the validity of the survey responses, any completed survey with greater than or equal to 2 inconsistent responses of the easiest sets of trade-off questions and choices that were only either to the right or left side on the survey were excluded. As the pair-wise rankings were consistent, PAPRIKA utilizes linear programming that analyzes the individual expert responses with the coefficient reported as the relative weights of the JAMRIS-SIJ measurement components [13]. The individual expert relative weights derived from the conjoint analysis were averaged for all experts to derive the relative weights for the JAMRIS-SIJ (Table 1).

### 2.2. JAMRIS-SIJ Vignette Ranking Exercise

Full profile magnetic resonance (MR) image + grade and a JAMRIS-SIJ grade-only vignette ranking exercise was performed to test the convergent and face validity of the partial-profile CAS-derived average JAMRIS-SIJ weights. All the participant experts were invited to complete the JAMRIS-SIJ grade-only vignette ranking, and a subset of twelve experts were additionally invited to complete the image + grade vignette ranking. The vignette ranking was completed before the CAS, allowing experts to rank the vignettes based on their prior expertise before being influenced by the effect of completing the conjoint analysis survey.

**Figure A3:**
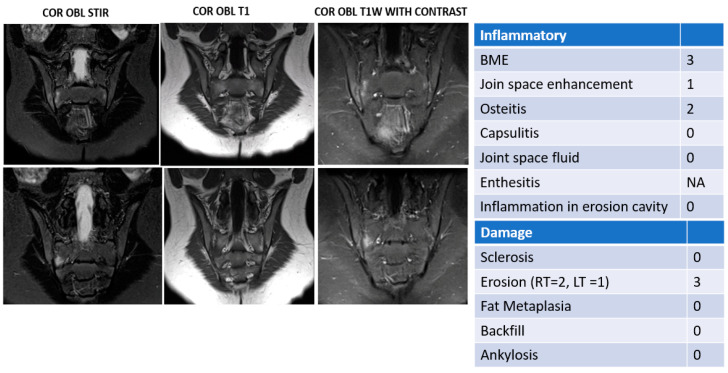
JAMRIS-SIJ image + grade vignette used for ranking exercise to test conjoint analysis survey (CAS)-derived weights. Fourteen image + grade vignettes were constructed corresponding to various combinations of pathologies in the JAMRIS-SIJ. Experts individually ranked the cases from the greatest to least level of inflammation and damage, respectively.

The MR image vignettes (Figure A3) are comprised of 14 bilateral SIJ-MRI studies represented by six coronal obliques MRI slices of T1-weighted (w), T2-w fat-suppressed (FS) or short tau inversion recovery (STIR), and T1-w FS post-contrast sequences to represent the SIJ pathologies according to the JAMRIS-SIJ scoring system [9]. Enthesitis was excluded from the inflammation domain measurement item for the image vignette ranking because the MR images provided did not include the optimized imaging planes for the assessment of enthesitis.

Twelve imaging experts in the imaging study cohort individually ranked the image vignettes in order of increasing severity or equivalence of inflammation or damage in the SIJ MR image. The vignettes were also scored using the JAMRIS-SIJ by consensus of three radiologists who did not participate in the ranking exercise to control confirmation bias. Two of the radiologists had more than 10 years of experience after training and one radiologist was in-training under the supervision of an experienced radiologist. Ten radiologists and two rheumatologists with experience in rheumatologic imaging completed the image + grade vignette ranking and constituted the imaging expert cohort (Figure A3 and Table S1). The MR image + grade vignette ranking was based on the individual items of the JAMRIS-SIJ system found on the MR images and reported by the JAMRIS-SIJ item grades, with a caution to avoid ranking the image vignette based on the composite of imaging findings to prevent obscuring the relative weights of each measurement item in arriving at the decision to rank order the vignette.

**Figure A4:**
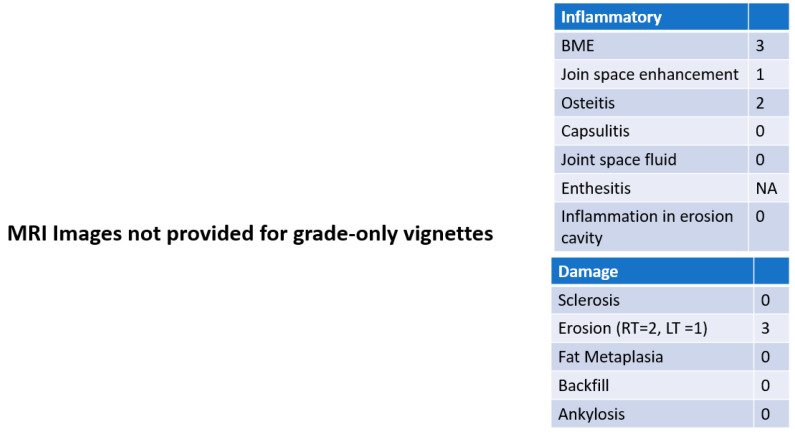
JAMRIS-SIJ grade-only vignette used for ranking exercise to test conjoint analysis survey (CAS)-derived weights. Fourteen grade-only vignettes were constructed corresponding to various combinations of pathologies in the JAMRIS-SIJ. Experts individually ranked the cases from the greatest to least level of inflammation and damage, respectively.

The JAMRIS-SIJ grade-only vignettes were prepared identical to the MR image vignettes, excluding the MR images. All imaging experts (except one radiologist who did not participate in the MR image + grade vignette ranking) completed the JAMRIS-SIJ grade-only vignettes (Figure A4 and Table S1). Participants were instructed not to change their JAMRIS-SIJ ranking after receiving the image vignettes as MR images were used to illustrate the scores, but instead, rank the image + grade vignettes separately. The weighted score for the 14 vignettes was derived by multiplying each expert’s CAS-derived weights by the vignette’s consensus JAMRIS-SIJ grades. The correlation of the CAS-derived JAMRIS-SIJ weights against the MR image + grade and JAMRIS-SIJ grade-only vignettes was tested. This correlation test cumulatively assessed the face, content, and convergent validities of the JAMRIS-SIJ relative item weights; the implicit item preference by PAPRIKA through transitivity of the adaptive partial profile CAS and the JAMRIS-SIJ.

### 2.3. Statistical Analysis

The absolute agreement among experts on their relative weights of the JAMRIS-SIJ in the CAS was assessed by calculating the two-way random single and average measure intraclass correlation coefficient (ICC) model 2,1 and 2,k (ICC 2,1 and 2,k) for grades above zero for each JAMRIS-SIJ item. Moreover, expert agreement in the vignette ranking exercise was assessed using the ICC, as described above [14]. The Spearman rank correlation was utilized to assess the correlation of the ranking of MR image + image vignettes and the JAMRIS-SIJ grade-only vignettes with CAS-derived weighted JAMRIS-SIJ ranking. For ICC interpretation, values ≤ 0.50 were defined as poor, 0.51–0.75 as moderate, 0.76–0.90 as good, and ≥0.91 as excellent reliability [14]. For the Spearman rank correlation coefficients, values ≤ 0.40 were defined as poor correlation, 0.41–0.60 as moderate, 0.61–0.80 as substantial, and ≥0.81 as high correlation. Statistical analysis was performed using the SAS software version 9.4 (SAS Institute Inc., Cary, NC, USA).

## 3. Result

### 3.1. Summary of Survey Items

A total of 153 potential item-grade combinations in the inflammation domain (Figure A1) and 90 in the damage domain (Figure A2) involving two JAMRIS-SIJ measurement items were possible in the survey, which were completed either explicitly by experts through pairwise comparison (Figure A1 and Figure A2) or implicitly through linear programming by the PAPRIKA algorithm 1000Minds software. The mean, range, and standard deviation (SD) of the number of item-grade combinations explicitly completed by experts was 35.6, 23–45, and 6.0 for the inflammation domain and 24.6, 17–28, and 2.7 for the damage domain, yielding 17 sets of relative weights unique to each expert. The average of the 17 relative weights from the experts was used as a template for the JAMRIS-SIJ weights (Table 1).

### 3.2. Conjoint Analysis Survey-Derived Relative JAMRIS-SIJ Weights

The average relative importance weights derived from the conjoint analysis survey had variable percentages depending on the grade and measurement items (Table 1). The relative weights for the highest grades among the inflammation items were osteitis (24.7%), bone marrow edema (24.3%), inflammation in erosion cavity (16.9%), joint space inflammation (13.1%), joint space fluid (9.1%), capsulitis (7.3%), and enthesitis (4.6%). Among JAMRIS-SIJ measurement items in the damage domain, the study average of the relative weights for the highest grades were ankylosis (41.3%), erosion (25.1%), backfill (13.9%), sclerosis (10.7%), and fat metaplasia lesions (9.1%). The complete set of average relative weights for the inflammation and damage domains is reported in Table 1.

### 3.3. Concordance of Conjoint Analysis Survey-Derived JAMRIS-SIJ Weights among Experts

The concordance of preference among the 17 experts in the conjoint analysis survey was moderate to excellent with ICCs for the inflammation domain survey of 0.60 (ICC 2.1) and 0.96 (ICC 2.k) (Figure A1 and Table 1) and damage domain survey of 0.73 (ICC 2.1) and 0.98 (ICC 2.k) (Figure A2 and Table 1). Item-wise agreement on the CAS-derived weights for JAMRIS-SIJ item grades ranged from 0.17 to 0.76 for the inflammatory domain items and 0.22 to 0.78 for the damage domain items (Table 1).

### 3.4. Homogeneity of Vignette Rankings (by Conjoint Analysis Survey (CAS) Score and by Explicit Expert Rank)

**Figure 1:**
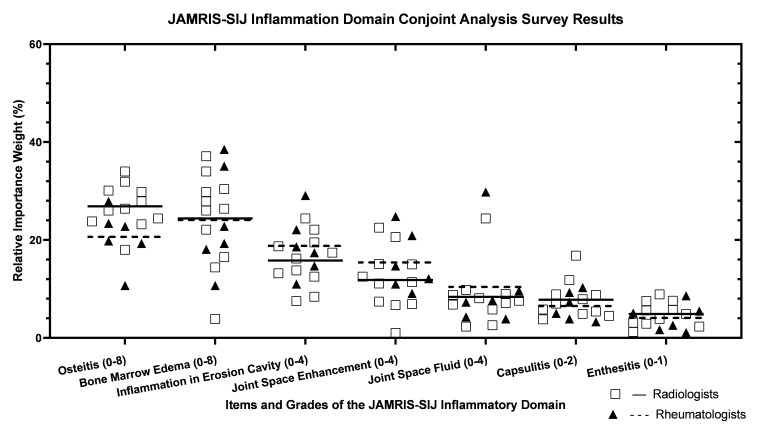

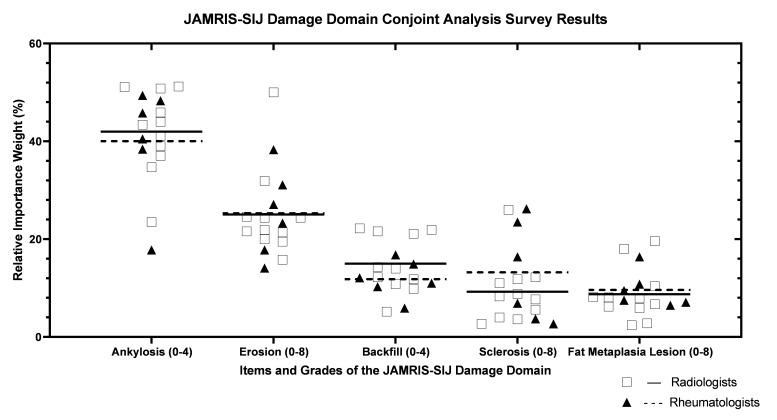
(**A**,**B**) Scatter plots of item weights derived from the conjoint analysis survey (CAS)-derived weights. (**A**) Scatter plots of inflammation domain item weights derived from the conjoint analysis survey (CAS)derived weights. Relative weights from all participants are plotted for each of the JAMRIS-SIJ inflammation domain items, with lines representing the median weight for radiologists (Square marker and solid line n = 11) and rheumatologists (triangular and broken line, n = 6). (**B**) Scatter plots of the damage domain item weights derived from the CAS-derived weights. Relative weights from all participants are plotted for each of the JAMRIS-SIJ damage domain items, with lines representing the median weight for radiologists (Square marker and solid line n = 11) and rheumatologists (triangular and broken line, n = 6).

**Figure 2:**
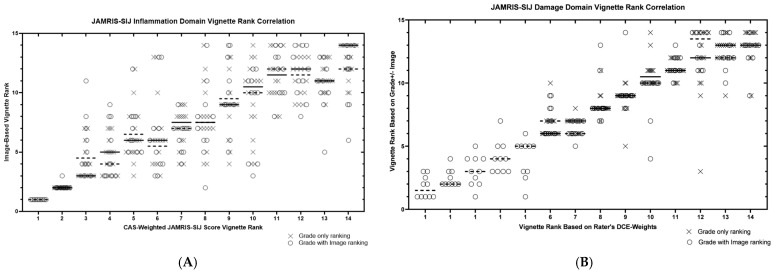
(**A**,**B**) Scatter plot of the of the JAMRIS-SIJ vignette ranks. (**A**) Scatter plot of the JAMRIS-SIJ vignette ranks produced by the full profile, MR image + grade ranking, ordered by consensus-graded, weighted JAMRIS-SIJ grade-only vignettes for the inflammation domain. Twelve imaging experts participated in the JAMRIS-SIJ image + grade ranking (circle maker), and 16 experts in the grade-only ranking (cross maker). For each of the 14 vignettes received, a consensus weighted grade rank on the x-axis and y-axis values for the image + grade ranks provided by the individual experts. Horizontal line denotes the median weighted score rank provided to each of the JAMRIS-SIJ MR image + grade vignettes. (**B**) Scatter plot of the of the JAMRIS-SIJ vignette ranks produced by the full profile, MR image + grade ranking, ordered by consensus-graded, weighted JAMRIS-SIJ grade-only vignettes for the damage domain. Twelve imaging experts participated in the JAMRIS-SIJ image + grade ranking (circle maker), and 16 experts in the grade-only ranking (cross maker). For each of the 14 vignettes received, a consensus weighted grade rank on the x-axis and y-axis values for the image + grade ranks provided by the individual experts. Horizontal line denotes the median weighted score rank provided to each of the JAMRIS-SIJ MR image + grade vignettes.

The homogeneity of the 14 CAS-weighted JAMRIS-SIJ vignette scores was observed in the vignettes with the least and most severe SIJ disease, with significant variability in cases with mild disease (Figure 1A,B). For the inflammation domain, the ICC (2,1) of weighted JAMRIS-SIJ vignette scores among the 17 experts was 0.80 when using the scores as ratio-level percentage data (i.e., 0–100%), and 0.87 when converting the percentages to ordinal-level rank data (i.e., 1–14). For the damage domain, the ICC (2,1) of the vignette scores among the 17 experts was 0.83 when using the scores as ratio-level percentage data, and 0.99 when using their ordinal-level rank data. Five case vignettes had no significant osteochondral damage findings present, hence all of them receiving all-zero grades as per the JAMRIS-SIJ definitions. Therefore, these five vignettes were indistinguishable by the JAMRIS-SIJ damage domain weighted score. Instead, they were separated by the vignette’s identification, ordered from the least to the greatest average grade-only vignette rank (Figure 2B).

The homogeneity of the 14 vignette ranks among the 16 experts by grade-only ranking was 0.83 for the inflammation domain, and 0.90 for the damage domain. For the imaging expert cohort, who also provided the concurrent grade + image ranking, the vignette ranks was 0.84 for the inflammation domain and 0.91 for the damage domain.

### 3.5. Correlation of JAMRIS-SIJ Vignette Ranking by MRI +/− Grade versus CAS Generated JAMRIS-SIJ Weights

**Figure 3:**
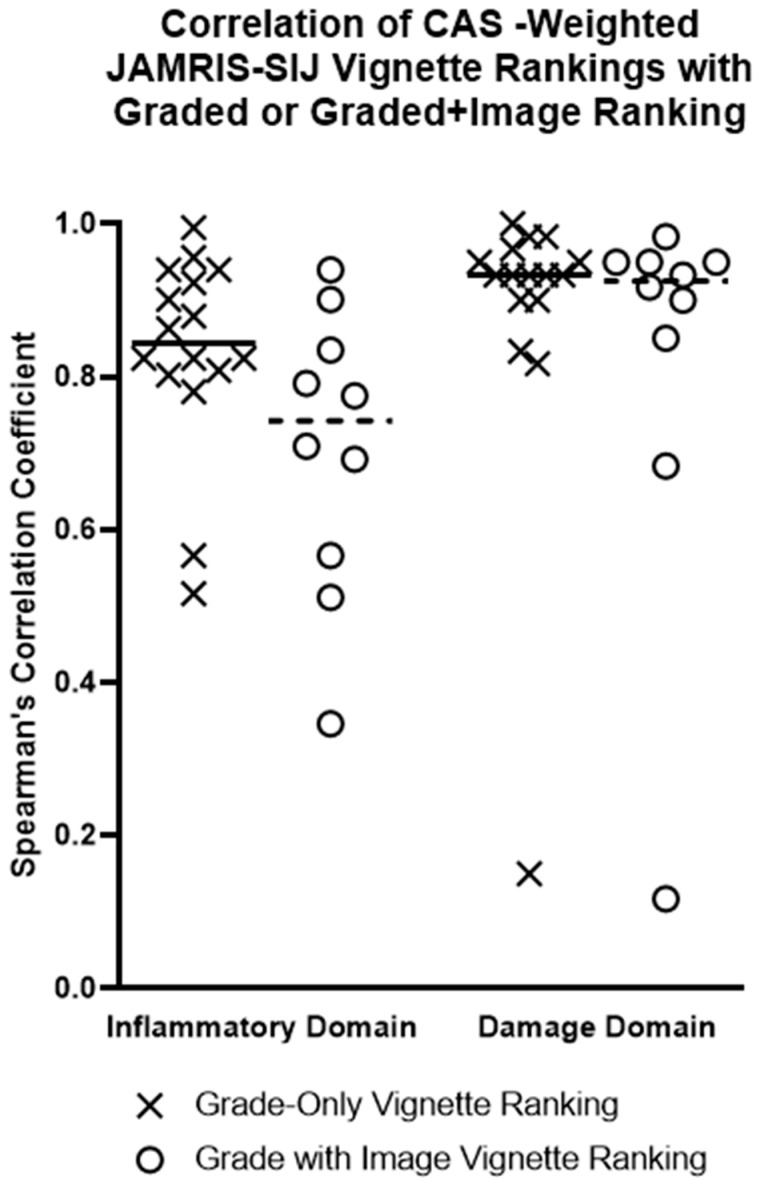
Correlation of CAS-Weighted JAMRIS-SIJ Vignette Ranking with Graded or Image Ranking. Spearman’s rank correlation coefficients are plotted for each expert rater (radiologist and rheumatologist, n = 17) comparing their two methods of producing of vignette ranks, i.e., correlating the experts’ grade-only or graded image-based full profile ranking with the ranking produced by applying the experts’ own CAS-derived weights applied to consensus grades. Horizontal lines represent the median Spearman correlation for each subgroup of participants (X—grade-only vignette ranking, n = 17, O—grade + image vignette ranking, n = 12). The JAMRIS-SIJ MR image + grade vignettes.

Of the 17 CAS-survey respondents, 16 participated in the vignette ranking exercise (Figure 2 and Figure 3). Each of the 16 experts provided two sets of vignette rankings, one produced by the expert’s independent ranking of grade-only vignettes and one produced by applying the expert’s CAS-derived weights to the grades (CAS weighted score rank). A subset of these experts (n = 12), who were imaging experts, also provided a third set of vignette rankings, derived using the grades of the vignettes as well as the representative MRI (“grade + image” ranking) to be correlated against their CAS-weighted score rank.

Correlation of the experts’ grade-only vignette rank with their CAS-weighted score rank showed a median Spearman correlation of 0.84 (IQR: 0.80–0.94) for the inflammation domain and 0.93 (IQR: 0.90–0.96) for the damage domain (Figure 3, Table 2). Subgroup differences were observed for this grade-only ranking between rheumatologists and radiologists (n = 6 and 10, respectively; Table 2). The correlation of the radiologists’ “grade + image” ranking against their CAS-weighted score rank showed a median Spearman correlation of 0.74 (IQR: 0.55–0.85) for the inflammation domain and 0.93 (IQR: 0.81–0.95) for the damage domain (Table 2).

## 4. Discussion

This study utilized a conjoint analysis of expert preferences to determine the relative weights of the measurement items within the JAMRIS-SIJ scoring system [15]. The experts in this study comprised eminent pediatric and adult rheumatologists and musculoskeletal radiologists with prior extensive experience in developing both adult and pediatric MR imaging scoring systems.

The relative weights of each grade for the inflammation and damage domain items are reported in Table 1. The two most important inflammation domain measurement items were bone marrow edema and osteitis, and their weights were equivalent (24.7% and 24.3%, respectively). Bone marrow edema and osteitis were 1.5 times more important than inflammation in erosion cavity, 1.9 times more important than joint space fluid, and 5.3 times more important than enthesitis. For the JAMRIS-SIJ damage domain, ankylosis was the most important measurement item, which was 1.6 times more important than erosion and 4.6 times relative to fat metaplasia, which was the least important item among the five measurement items in the JAMRIS-SIJ damage domain. Erosion was rated second in relative importance by expert preference among the JAMRIS-SIJ damage domain, having 1.8 times more importance than backfill and 1.3 times more importance compared to sclerosis. The differential weights of the measurement items in the JAMRIS-SIJ are similar to the MRI scoring system for temporomandibular joint in JIA [16].

The presence of ankylosis signals advanced disease, which is in most cases a hallmark of irreversible osteochondral damage, while bone marrow edema and erosion are the preferred measurement items in discriminating the response to intervention in patients with JIA since these can be reversible entities, providing an objective metric for clinical decision making. The presence of bone marrow edema is indicative of active disease, providing clinical evidence for the initiation of therapy. Likewise, erosion has been reported to be a negative prognostic factor that warrants the use of more aggressive therapy in JIA, such as biologic agents [17].

The clustering of the relative weights of items in the inflammation domain was homogenous for both radiologists and rheumatologists, with a greater variability for bone marrow edema and joint space fluid. In the damage domain, there were substantial outliers of expert preference across all measurement items. This may be related to the complexities of the damage domain item definition and interpretation, further than in adults’ occurrence of sacroiliitis, and resulting in part in the infrequent presence of the damage domain items among JIA patients. It may also be due to the availability and access to advanced imaging with prompt intervention that limits the progression of the JIA disease course to osteochondral damage.

Trends for the item weights at lower grades were not consistent with the highest grade weights. In the inflammation domain, inflammation in the erosion cavity at grade level 1 was 5.3%, which was higher in the point value than osteitis (3.9%) and bone marrow edema (3.2%). This was similar for joint space enhancement (4.5%) and capsulitis (4.6%). There were similar nonlinear trends in the intermediate grades in the inflammation domain items. However, in the damage domain, except for backfill, the item weights were consistent between grades. These grade-related differences in the JAMRIS-SIJ measurement items are likely due to the expert misperception on their preferences of SIJ MR imaging pathology at lower grades compared to higher grades among the measurement items. For example, the presence of bone marrow edema measured in 0–8 grades for a single quadrant of the SIJ MR image may suggest lesser disease compared to the presence of inflammation in an erosion cavity, which is measured in 0–4 grades in the superior half of an SIJ MR image. The differences in the measurement item levels of grades, for a four-grade level item, such as in the case of inflammation in erosion cavity, compared to an eight-grade level item, such as in the case of bone marrow edema, may distort the expert perception of the severity of the patient scenario of either inflammation or damage in the partial profile provided in the conjoint analysis survey. This may have resulted in scenarios where the experts have disproportionately weighted more disease for 0–4 grade level items and lesser disease for 0–8 grade level items.

The differences in the measurement value of osteitis and bone marrow edema are yet to be ascertained, as both measurement items are of uncertain origin that signal active disease and have similar clinical response to treatment. However, osteitis requires contrast enhancement for visualization, which is a significant limitation for its use in pediatric imaging due to the concern of contrast accumulation in the central nervous system after multiple scans and nephrogenic systemic fibrosis in patients with limited renal function [18,19]. Bone marrow edema may be present due to other causes unrelated to JIA, such as mechanical overload, trauma, infection, and neoplasm. In a recent study, bone marrow edema-like lesions were shown due to the normal variability in subchondral bone marrow signal in growing children [20]. Whether osteitis and bone marrow edema should both be scored would sensibly depend on the imaging protocol used.

Determining whether a small amount of fluid in the joint space is pathologic becomes challenging when associated pathologic findings, such as bone marrow edema, are absent. Correlations found between joint effusion and bone marrow edema measurement items may be partially due to their pathogenesis. This could have contributed to the experts’ preferences for relative weights of individual items. Further studies are needed in the future to improve our understanding on the inter-relationship of items which may help us reduce the variance among experts in allocating point values for individual measurement items.

Imaging outcome measurement tools are increasingly used to assess intervention effectiveness in musculoskeletal disease clinical trials, with evidence supporting the use of MRI as the preferred modality of choice in JIA [21]. To objectively assess JIA disease activity and change after therapeutic intervention, it is possible to use the multi-component JAMRIS-SIJ score without weighting each component. However, it is also desirable to generate a single composite score as a summary biomarker. If a single score is to be generated, this requires the relative weighting of its measurement items for clinical importance in some way. Ideally, this will improve the construct validity of the composite domain score by increasing the weighing of changes which are more specific and/or sensitive to JIA.

This study has some limitations. Chief among them is the expert-driven weighting method used. Experts’ preferences vary considerably due to the expected differences among the patient population and in clinical experiences. The validity of experts’ preferences was also limited by subject matter expertise, a criterion that is not impervious to fallibility. Moreover, the differential preferences of experts did not necessarily account for the inherent intercorrelation of the measurement items, for which there are gaps in the knowledge base in the literature. Furthermore, the sampling of the JAMRIS-SIJ full profile image vignettes was limited in this study as we tried to minimize the pragmatic issues related to survey fatigue that could have arisen from the assessment of a large sample of imaging vignettes at a single setting. This sample size limitation in this study may have influenced the coefficients of correlation of the JAMRIS-SIJ weighted scores and the JAMRIS-SIJ vignette ranking.

## 5. Conclusions

This study used a formal conjoint analysis-based survey to elicit expert preferences on the relative weights of measurement items and grades, which are necessary to generate single summaries for the two domains of the JAMRIS-SIJ from constituent items. The face and content validity of the partial profile CAS-derived JAMRIS-SIJ weights was high when compared to the full profile vignettes defined by grades with and without representative images. These weights may provide value, by helping to appropriately measure disease activity and treatment effectiveness in JIA clinical trials.

## Figures and Tables

**Table 1:** Conjoint analysis survey-derived relative percentage weight for the measurement components of the JAMRIS-SIJ. Following the grading of an image, the percentage weight of each component grade was added to constitute the domain percentage disease severity score ranging from 0–100% for seven inflammation and five damage domain items, respectively. The percentage weights are reported as group means relative weights. BME; bone marrow edema, IEC; inflammation in erosion cavity, JSE; joint space enhancement, JSF; joint space fluid, FML; fat metaplasia lesion. ICC: intraclass correlation coefficient.

Inflammation Domain											
	Grades										
	0	1	2	3	4	5	6	7	8	ICC (2,1)	ICC (2,k)
Osteitis	0	3.9	7.7	11.2	14.4	17.2	19.8	23.3	24.7	0.66	0.97
BME	0	3.2	6.4	9.5	12.6	15.6	18.5	21.4	24.3	0.59	0.96
IEC	0	5.3	10	13.7	16.9	--	--	--	--	0.52	0.95
JSE	0	4.5	8.2	10.9	13.1	--	--	--	--	0.34	0.90
JSF	0	2.8	5.1	7.1	9.1	--	--	--	--	0.17	0.78
Capsulitis	0	3.9	7.3	--	--	--	--	--	--	0.41	0.92
Enthesitis	0	4.6	--	--	--	--	--	--	--	0.76	0.98
All Items	--	--	--	--	--	--	--	--	--	0.60	0.96
**Damage Domain**											
	**Grades**										
	**0**	**1**	**2**	**3**	**4**	**5**	**6**	**7**	**8**	**ICC (2,1)**	**ICC (2,k)**
Ankylosis	0	10	20.2	30.7	41.3					0.79	0.98
Erosion	0	3.7	7.2	10.6	13.9	16.9	19.8	22.5	25.1	0.60	0.96
Backfill	0	3.9	7.5	10.8	13.9					0.59	0.96
Sclerosis	0	1.8	3.6	55.2	6.6	7.8	8.9	9.8	10.7	0.23	0.83
FML	0	1.6	3.1	4.5	5.7	6.6	7.5	8.3	9.1	0.33	0.90
All Items										0.73	0.98

**Table 2:** Spearman’s correlation coefficients of four different datasets. Correlation of experts’ preference for JAMRIS-SIJ measurement item weights for MR image, JAMRIS-SIJ score vignettes and a combination of MR image and score vignettes. CAS—conjoint analysis survey IQR—interquartile range.

		Spearman Correlation Coefficient of CAS-Weighted JAMRIS-SIJ vs. Graded ± Image Vignette Ranking			
Vignette Type	Expert Cohort	Inflammation Domain		Damage Domain	
		Median	IQR	Median	IQR
Grade only	All experts (n = 17)	0.84	0.80–0.94	0.93	0.90–0.96
	Rheumatologists (n = 6)	0.89	0.82–0.97	0.92	0.88–0.95
	Radiologists (n = 11)	0.82	0.73–0.93	0.94	0.91–0.97
Grade + Image	Radiologists (n = 10) Rheumatologist (n = 2)	0.74	0.55–0.85	0.93	0.81–0.95

## Data Availability

The data presented in this study are available on request from the corresponding author. The data are not available publicly due to privacy and ethical reasons.

## Supplementary Materials

The following supporting information can be downloaded at: https://www.mdpi.com/article/10.3390/jcm12072729/s1, Table S1: Distribution of individual experts with the years of experience into imaging and clinician expert cohort. The imaging expert cohort comprises of ten radiologist and two rheumatologists with imaging interpretation experience who ranked the JAMRIS-SIJ image+grade and grade-only vi-gnettes. The clinician expert cohort comprise of one radiologist and 4 rheumatologist who com-pleted only the JAMRIS-SIJ grade-only vignettes.
