# Supplementary material for: Determination of Relative Weightings for Sacroiliac Joint Pathologies in the OMERACT Juvenile Arthritis Magnetic Resonance Imaging Sacroiliac Joint Score
Source: J Clin Med. 2023 Apr 6;12(7):2729. doi: 10.3390/jcm12072729 (PMC10095587; doi:10.3390/jcm12072729)
Supplement: Supplementary file 1 [file jcm-12-02729-s001.zip › jcm-2197064-supplementary.pdf]

Table S1. Distribution of individual experts with the years of experience into imaging and clinican expert cohort. The imaging expert cohort comprises of ten radiologist and two rheumatologists with image interpretation experience who ranked the JAMRIS-SIJ image+grade and grade-only vignettes. The clinician expert cohort comprise of one radiologist and 4 rheumatologist who completed only the JAMRIS-SIJ grade-only vignettes.

Expert ID	Expert Specialty		Expert Years of Experience					Imaging Expert Cohort (Image+JAMRIS-SIJ Vignettes) N=12	Clinician Expert Cohort (JAMRIS-SIJ Vignettes) N=5
	**Radiology N=11**	Rheumatology N=6	1-4	5-10	11-15	16-20	21-30		
**65600**	×		×						×
**65807**	×		×					×	
**59787**	×			×				×	
**62564**	×			×				×	
**60522**		×		×					×
**60694**		×		×					×
**63454**		×		×					×
**59786**	×				×			×	
**64357**	×				×			×	
**63385**	×					×		×	
**64364**		×				×			×
**64541**		×				×		×	
**64805**		×				×		×	
**63574**	×						×	×	
**63575**	×						×	×	
**68684**	×						×	×	
**64693**	×						×	×	
